# SPOUSAL COMBINATIONS OF EMPLOYMENT STATUS AND SHARED HEALTH RISKS AMONG OLDER MARRIED COUPLES

**DOI:** 10.1093/geroni/igae098.0557

**Published:** 2024-12-31

**Authors:** Ha Young Choi, Shannon Mejia

**Affiliations:** University of Illinois Urbana-Champaign, Champaign, Illinois, United States; University of Illinois Urbana-Champaign, Urbana, Illinois, United States

## Abstract

Continuing employment has been shown to protect health into old age. However, within marriages, the decision to retire or work is jointly negotiated which may be differentiated by socioeconomic resources. This study explores how spousal combinations of employment status differentiate individual and shared manifestations of cumulative health risk. Utilizing data of 3,143 older couples (aged 51-79) from the 2008/2010 Health and Retirement Study, we first conducted latent class analysis of husbands’ and wives’ combined employment statuses (age range fully/partially working: 51-79). We identified five classes of spouses’ combined statuses: (1) one unemployed, another not fully working (8.15%), (2) retiree couples (34.36%), (3) one unemployed, another fully working (3.63%), (4) one fully working, another partially working (45.28%), and (5) fully working couples (8.59%). Latent dyadic models parsed the outcome variables, cumulative indicators of physiologic reserve and metabolic syndrome, into individual and shared components, which were then regressed on predicted cluster membership, and adjusted by age and functional limitations. Physiologic reserve was lowest in the one underemployed/other fully working class and highest in both fully working class. Meanwhile, systematic differences in metabolic syndrome across classes were only observed in couples where neither spouse had attended some college. In these couples, metabolic syndrome was highest in the two classes where either spouse was partly or fully working. Our findings imply that the effects of extended work life on health risks are contextualized within marriage and socioeconomic resources, where the shared health of couples in lower socioeconomic status rely more on combined employment status.

